# Shared Care Practices in Community Addiction and Mental Health Services: A Qualitative Study on the Experiences and Perspectives of Stakeholders

**DOI:** 10.3390/healthcare10050831

**Published:** 2022-04-30

**Authors:** Michele Foster, Julia Weaver, Reham Shalaby, Ejemai Eboreime, Kimberly Poong, April Gusnowski, Mark Snaterse, Shireen Surood, Liana Urichuk, Vincent I. O. Agyapong

**Affiliations:** 1 Department of Psychiatry, Faculty of Medicine, University of Alberta, Edmonton, AB T6G 2B7, Canada; foster@ualberta.ca (M.F.); rshalaby@ualberta.ca (R.S.); eboreime@ualberta.ca (E.E.); liana.urichuk@albertahealthservices.ca (L.U.); 2 Alberta Health Services, Addiction and Mental Health, Edmonton, AB T5J 0G5, Canada; julia.weaver1@gmail.com (J.W.); kimberly.poong@ahs.ca (K.P.); april.gusnowski@albertahealthservices.ca (A.G.); mark.snaterse@albertahealthservices.ca (M.S.); shireen.surood@albertahealthservices.ca (S.S.); 3 Department of Psychiatry, Faculty of Medicine, Dalhousie University, Halifax, NS B3H 2E2, Canada

**Keywords:** addiction and mental health, shared care, stakeholders

## Abstract

Shared care involves collaboration between primary care, secondary and tertiary care that enables the allocation of responsibilities of care according to the treatment needs of patients over the course of a mental illness. This study aims to determine stakeholders’ perspectives on the features of an ideal shared care model and barriers to practicing shared care within addiction and mental health programs in Edmonton, Canada. This is a qualitative cross-sectional study with data collected through focus group discussions. Participants included patients, general practitioners, psychiatrists, management, and therapists working in primary and secondary addiction and mental health. Responses were audio-recorded, transcribed, and analyzed thematically. Perceived barriers to the implementation of an ideal shared care model identified by participants include fragmented communication between primary and secondary healthcare providers, patient and family physician discomfort with discussing addiction and mental health, a lack of staff capacity, confidentiality issues, and practitioner buy-in. Participants also identified enablers to include implementing shared electronic medical record systems, improving communication and collaboration, physical co-location, and increasing practitioner awareness of appropriate referrals and services. This original research provides stakeholders’ perspectives on the features of an ideal shared care model and barriers to practicing shared care within addiction and mental health programs.

## 1. Introduction

According to the Canadian Mental Health Association, in any given year, 20% of Canadians will experience a mental health problem or illness. However, previous research shows that just 35% of those individuals will seek treatment [1]. Primary care providers, or family physicians, are often the first point of contact for individuals with mental health and addictions concerns [2]; as a result, according to the World Health Organization, primary care should be seen as an integral part of mental health care delivery [3]. A significant link exists between physical and mental health. Poor physical health, for example, is a potential risk factor for developing depression, while depression is a risk factor for a number of adverse health conditions, including cancer, cardiovascular disease, stroke and diabetes. As a result of interacting physical and mental health concerns, people with schizophrenia or bipolar disorders can experience a reduction in life span of up to 20 years [4,5,6]. Moreover, certain medications used to treat severe mental illnesses are associated with adverse physical health outcomes, often leading to increased healthcare system utilization [5,7,8]. 

Accordingly, there is a growing need for a shared care model connecting physical and mental healthcare providers. Generally, shared (or collaborative) care refers to the collaboration and shared responsibility between providers in tertiary, secondary and primary care. This collaboration sometimes extends to include providers, health care leaders, policy planners, and community participation [5,9,10]. Shared care models are practiced by different specialties and for the management of common physical health ailments, including ophthalmology [11,12], pediatrics [13,14], leukemia [15], and diabetes [16,17,18]. In the mental healthcare delivery system, one definition of shared care is: “a process of collaboration between the family physician and the psychiatrist that enables the responsibilities of care to be apportioned according to the treatment needs of the patient at different points in time in the course of a mental illness and the respective skills of the family physician and psychiatrist” [18]. 

In Canada, a shared care initiative was established in 1996; this change was facilitated by the position paper which was approved by both the Canadian Psychiatric Association and The College of Family Physicians of Canada [18]. Since then, the model has grown in popularity, from isolated initiatives to being adopted by regional and provincial health authorities [5,18].

Shared or collaborative care in mental health and primary health care settings is thought to decrease the burden of illness experienced by individuals with a mental illness by optimizing their care and increasing access to mental health services and mental health promotion [19]. Shared mental health care is not an alternate style of practice; rather, it can become a valuable extension of the current clinical practices of psychiatrists and family physicians, enriching the care that each is able to offer [18]. When considering the relationship between family physicians and mental health care providers, it is important to reduce fragmentation and achieve integration of mental health services [20]. The organization of services and their integration into primary care, which is usually overburdened with other health priorities, is a significant barrier to the expansion of mental health services [4]. 

There are several studies looking at the implementation of shared or collaborative care in mental health [21,22], but only a small number have looked at stakeholder perspectives [23]. One example is Ion and colleagues’ exploration of provider and client experiences of integrated care in Ontario. These stakeholders opined that the overarching concept of integrated care include co-location of care; continuity of care; team composition and functioning; client centeredness; and comprehensive care for individuals and populations [24]. Kroenke and Unutzer, having examined comprehensive reviews of the literature, identified six key components of shared care. These include a population-based approach, measurement-based care, treatment to target strategy, care management, supervision by a mental health professional, and brief psychological therapies [21]. While Evans et al. opine that multidisciplinary teams are essential to integrated care, the diverse perspectives from the literature suggest that a contextual definition of shared care is essential for the various care settings.

This paper adds to the literature by exploring perspectives of a range of professionals involved in the delivery of care, as well as providing a contextual perspective of an ideal model. Our qualitative study examines the perceived barriers to the implementation of shared care models within addiction and mental health programs in the Edmonton zone of Alberta, Canada. The specific objective of our study is to determine stakeholders’ perspectives on the features of an ideal shared care model and the barriers to the practice of shared care within addiction and mental health programs in the Edmonton zone. Our research questions include:

1. What are stakeholders’ perceptions of what a shared care model within addiction and mental health should look like?
2. What are stakeholders’ perceptions of barriers to the practice of shared care within addiction and mental health programs in the Edmonton zone?

### Conceptual Framework

**Figure 1:**
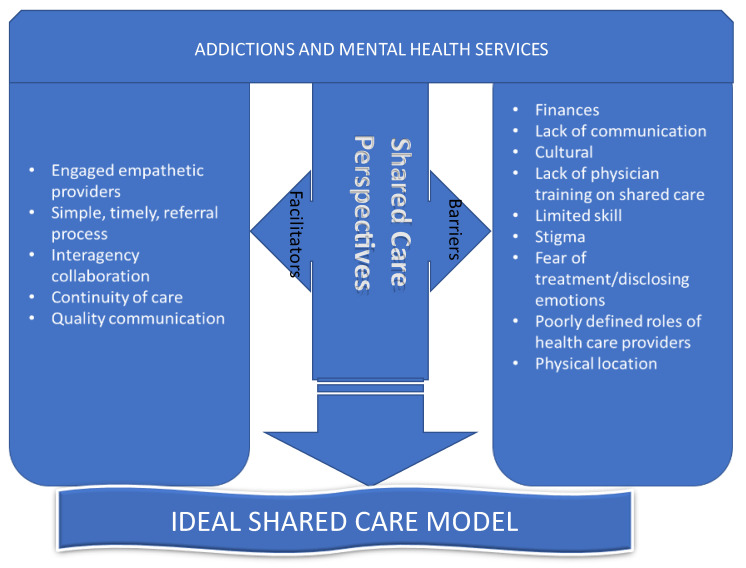
Conceptual framework of an ideal shared care model.

A conceptual framework brings together a number of related concepts to explain or predict a given event or give a broader understanding of the phenomenon of interest [25]. We developed a conceptual framework for our study based on evidence from the literature, which suggests that there are positive or facilitating factors, but also significant barriers to the successful implementation of a shared care model within addiction and mental health care programs [9,18,26,27,28,29,30,31,32,33,34,35,36], as illustrated in Figure 1. 

## 2. Materials and Methods

### 2.1. Study Setting 

The study was conducted within the Edmonton Zone, an administrative health region in Alberta with a population of 1,321,426, according to the 2016 census [37]. Primary addiction and mental health services in Alberta are provided predominantly through Primary Care Networks (PCNs) and MediCentres. The latter provide mostly General Practitioner (GP)-based walk-in services with limited allied mental health care, while most PCNs have GPs working closely with Behavioural Health Consultants who provide short term psychological support for patients. Some PCNs have visiting psychiatrists who provide one-time or initial consultation services for clients attending the PCN who have been referred by their GP. Additional independent or standalone primary care GP practices and privately-run psychological services are also available. Secondary and tertiary addiction and mental health services in Alberta are provided predominantly by Alberta Health Services and Covenant Health (the largest Catholic health care provider in Canada), although there are also a number of private psychiatrist clinics providing secondary mental health consultations and follow-up care. 

### 2.2. Data Collection

Study participants included purposively selected staff working with addiction and mental health clients in primary or secondary care in the community, as well as clients accessing primary and secondary addiction and mental health services.

KP, AG and MF (who have expertise in mental health and health services management) conducted nine focus groups between 12 January and 9 March 2018, and talked to 56 people, including: 13 patients, 3 physicians, 14 managers, and 5 psychiatrists from Alberta Health Services (AHS) addiction and mental health (AMH), and 9 managers, 7 family physicians, and 5 therapists from Primary Care Networks (PCNs).

Participants were engaged in one-to-two-hour discussions guided by a series of questions which were adapted slightly based on which service provider or client group was present. These included questions about: patient/client comfort with discussing addiction and mental health concerns with their doctor, family doctors’ experiences with managing addictions and mental health symptoms, the current relationship between AHS AMH and primary care, the ideal relationship between AHS AMH and primary care, what shared care means to participants, and factors that enable and prevent shared care between AHS AMH and primary care. Data saturation was determined to have been attained when no new information was received from further focus group discussions.

### 2.3. Data Analysis

Participants’ discussions on the questions were recorded, transcribed and analyzed thematically using NVivo 12 software. Inductive thematic analysis was conducted by two qualitative analysts (JW and MF), guided by the steps described by Braun and Clarke [38]. First, the analysts familiarized themselves with the data by listening to the recordings, reading through the transcripts and taking initial notes. Thereafter, initial coding was conducted, and observed patterns were used to generate themes. These themes were reviewed and harmonized by both analysts together. Our analysis and reporting followed the qualitative description approach. This approach, focuses on discovering the who, what, and where of events or experiences and gaining insights from informants regarding a poorly understood phenomenon.

## 3. Results

### 3.1. Defining Shared Care

Definitions and descriptions of shared care differed widely across conversations with stakeholder groups. One common emphasis was that shared care models should be client-centered, although the stress placed upon this varied. Participants noted that therapists and other professionals play a central role in shared care and that they should be a core part of any shared care model. However, individuals disagreed about how shared care should be structured, with some envisioning a short-term consultation model and others envisioning long-term involvement from psychiatrists and addiction and mental health (AMH) specialists. Participants also described opposing expectations of the roles of care practitioners and contradictory understandings of collaborative care as a fundamental barrier to implementing shared care in and of itself.

AHS AMH Management: “Our private doctors work very differently, and clinics work very differently and it’s—how do you begin to standardize that understanding of shared care, right”?

#### 3.1.1. Client-Centered Care: “The Patient Gets What They Need When They Need It”

Participants from all groups posited that clients’ needs and goals should be at the center of any shared care model. They emphasized that clients should feel able to trust their care providers, have input in their treatment, receive consistent messaging from their care providers, and know what information will go to whom and who to approach with different concerns. Despite this, many participants’ contributions focused solely on clinician and physician roles, not on the client’s experience, indicating discrepancy about the perceived importance of client-centeredness as a principle of shared care.

AHS AMH Clinician: *“I don’t know how many times we get calls from clients where they’re like, ‘Well this person told me this and this person told me this, and this person told me this.’”*

AHS AMH Management: *“Sometimes patients and families are treated like a hot potato, right? Well, they’re not severe enough for this program, so we’ll ask them to go here, but this program feels that their issues that they’re presenting with are more than what they can handle, and this poor family or client is kind of stuck in the middle, right.”*

#### 3.1.2. A Holistic Team Approach: “Having a Team Is Essential”

Many participants described a team-based approach to care involving many different care providers, including mental health therapists, occupational and recreational therapists, addictions counsellors, nurses, nurse practitioners, dieticians, psychologists, case managers, independent living support (ILS) workers, and social workers. Some participants’ descriptions of the importance of these non-physician roles were in response to the physician-centric definition of shared care provided in the focus groups. Team members with diverse specialties were seen as integral to providing holistic shared care for clients. However, participants also noted other benefits to having these care providers as a part of a shared care model:

- Reduced load on overburdened psychiatrists and family physicians who work within a fee-for-service model that incentivizes seeing more patients in less time.
- The ability to use therapeutic approaches other than medication, potentially preventing the need for more psychiatric interventions.
- Especially in regards to social workers, case managers, and Independent Living Support (ILS) workers, helping clients navigate the mental health and social service systems and acting as a key connection point between other team members.
- Ongoing support and education around mental health and addictions for family physicians.

PCN Management: *“It’s a flawed belief that just the doctor can fix […] I think it needs a whole team around that.”*

Family Physician: *“Yeah, we’re not mental health therapists, right? And many people, that’s what they need, they don’t need medication, they need CBT, they need support, they need … they can be kept from getting on medication if they have access to people that actually are really good at providing those kinds of treatments.”*

Participants remarked that to make this type of team approach work effectively, there must be clarity about scope of practice and roles in shared care.

PCN Management: *“I think the shared care requires a really good understanding of the other providers. You’ve got to know where the overlap is, where the handoffs are appropriate, and so there’s a big part of that common understanding and maybe a lot of it with the family physicians and psychiatrists because they’re coming from the same discipline, but you start to throw in mental health therapists, your health consultants and RNs, social workers, you name them, […] we’re not sure where one’s role begins and the other ends, and I think that’s vital.”*

#### 3.1.3. Models of Shared Care: “There’s Different Ways of […] Doing Shared Care”

Participants held differing views about the ideal structure of a shared care model. In particular, psychiatrists disagreed with one another about the type of role they should have in shared care. For example, some participants placed more responsibility on family physicians, with psychiatrists as short-term consultants, while others envisioned more long-term psychiatrist or addictions and mental health specialist involvement.

Participants (mainly psychiatrists) who argued for formal short-term consultation models emphasized limited psychiatric resources and the need to reduce wait times for psychiatric care. They stressed that family physicians should take over follow-up care with clients after a one-time or limited psychiatric consultation.

Psychiatrist: *“So let’s say we have enough family doctors, then it’s going to be much easier for us to kind of see a patient, give an outline of say, give this medication, give that, and if it doesn’t work after say two three four weeks, try this, try that […]. If that doesn’t work, then the patient can be sent back to me.”*

Some participants noted the benefits of family physicians having short, informal consultations (either phone calls or in-person) with addictions and mental health specialists and psychiatrists. Participants who advocated for this model identified a need for rapid access to specialized help and advice, such as phone-based guidance.

Family Physician: *“Guidance, I totally agree. Support, or in my way of saying is guidance. Somebody on the phone, or somebody tell us exactly these are the steps to be followed so we don’t have to actually refer the patient down to the addiction center […] I think, as long as we have some guidance, we have some support from the right authorities, I think why not. I think we are great and in a much better position to do that than a third party”.*

Some participants who advocated for short-term consultation agreed that not all clients could be served by one-time consults and that long-term involvement might sometimes be necessary. Other advocates for long-term psychiatric involvement argued that psychiatrists should manage clients until they are stabilized, not just for a predetermined number of visits. Some criticized the current practice of requiring doctors and community practitioners to re-refer clients after consultations or short-term therapeutic interventions, resulting in longer wait times.

Psychiatrist: *“But because mental health and addictions are longitudinal problems, I think there is a role to ensure that our part of the system stays involved in a longitudinal way. And then it doesn’t have to be a re-referral back in with, you know, a one month or two month long wait before they can access a program that had clearly benefitted them before.”*

Family Physician: *“It is doing the assessment, it’s seen by the psychiatrist, and it is being followed up on a regular basis until the patient is now stable enough to be discharged back to your practice.”*

Participants who advocated for either model often put emphasis on family physicians taking on long-term follow-up care for clients, either until stabilization, after a psychiatrist has assumed care for a period of time, or after a one-time consult.

PCN Management: *“Addiction or mental health is an ideal problem for the community and family physicians because it’s long-term, and you need that team, so no different than having the diabetic in a practice forever if you look at it, sure, you want to get them to be not addicted and all that, but it is chronic, and so it fits nicely in family medicine because there’s already a long-term support in place if the skills are there.”*

Psychiatrist: *“I think [family physicians] should be very actively involved in follow-up […] I think with collaboration and help from us, I think they should be following up with patients. I think a lot of family doctors are reluctant to do so, they’re quite happy for us to take over, but I think we really need to push and explore more collaboration with primary care in relation to follow up with patients.”*

### 3.2. Obstacles to Shared Care

Participants noted a number of barriers to instituting shared care, including a lack of communication between addictions and mental health services and family physicians, family physician barriers, a lack of addictions and mental health capacity, legal concerns including issues with confidentiality, and practitioner buy-in.

#### 3.2.1. Fragmented Communication: “Like an Estranged Marriage”

Many participants said they had no relationships or communication between primary care and addictions and mental health services. Some participants also mentioned specific contributors to this communication barrier, including staff turnover, inconsistent and fragmented communication during transitions (e.g., referral and follow-up), and a historic practice of not communicating.

Family Physician: *“To me it feels a bit vacuous because I know they’re out there, I know my patients are sometimes having contact with the services, and I feel like we don’t have any kind of back and forth communication about what that is, and we’re not a team working for this patient’s best interest together, that it’s kind of just these two disparate interactions with no coordination.”*

Participants’ current standard communication practices were limited to minimal contact such as sending letters to acknowledge referrals, initial assessments, file closures, and transitions from inpatient care. Overall, participants acknowledged the need for AMH services to improve their communication and timely follow-up with all community providers, including physicians, and use communication methods beyond a faxed initial consultation letter.

Family Doctor: *“If you send someone to a private psychiatrist, you’ll get a consult letter back but often not follow up visits or maintenance visits or anything like that.”*

PCN Therapist: *“I don’t know if it’s everywhere, but most of the time the therapist at [clinic name] or [clinic name] will send the first letter once they’ve connected with the family doctor’s patient to the family doctor saying “I’ve seen this person, this is what we’re going to work on,” and then they never hear from them again.”*

#### 3.2.2. Family Physician Barriers: “Are the Doctors Comfortable Discussing Addictions and Mental Health Concerns?”

Participants noted a number of inter-related primary care barriers, including: clients’ discomfort with discussing mental health or addictions with their doctors; family physicians’ discomfort and lack of knowledge about addiction and mental health; and the effect of the fee-for-service compensation model on physicians’ ability to address AMH issues.

Many participants identified that stigma can make clients less comfortable talking to their family doctor about mental health and addictions. Others mentioned that some clients might not consider discussing AMH issues with their doctor or expressed that clients may not want a family doctor for similar reasons. Some participants who were patients expressed concerns that clients with addictions might be even less likely to talk to their physician because of a fear that it would affect their care or prescriptions (e.g., being labelled as “pill-seeking”). Finally, some participants noted that their clients did not have a family doctor in the first place.

Client: *“I wouldn’t feel comfortable talking it over with my family doctor, no.”*

AHS AMH Management: *“I still think stigma is a big, big one that from a patient’s perspective that I would much rather compartmentalize my own care. […] what you’re telling me here is no, no that’s wrong, that the psychologist should be sharing my horrible thoughts with my family doctor that takes care of my kid, treats their foot fungus, and now you’re going to be talking about that? I think these are confidentiality and disclosure, and it’s tied with a bit of stigma and personal baggage.”*

Participants mentioned that clients’ discomfort with family doctors may also be a reflection of family doctors’ own discomfort with AMH issues; these participants emphasized that many physicians may hold stigmatizing views about AMH and/or may not have the education or confidence to address these concerns comfortably with their patients.

Some participants noted that while most doctors are able to address mild or moderate depression and anxiety, they might not have the ability or desire to treat more severe, complex, or stigmatizing mental health and addiction concerns. These participants called for more education and resources for family physicians, including training on resources and therapeutic approaches. Several participants noted that even simply getting family doctors to ask patients about mental health and addiction during regular visits would help make clients feel more comfortable.

Family Physician: *“I know I have a lot of colleagues who don’t want it to come up because they don’t feel like they have solutions or things that they can do if these sorts of issues arise, and so they’ll like almost deliberately skirt the issue and like, “let’s deal with your knee! Please don’t cry!” So that they don’t have to deal with this when they have no solutions or no ideas about what to do if a mental health concern comes up.”*

PCN Therapist: *“We have a lot of walk-in clinics and we get the impression on the mental health team that if somebody sheds one tear it’s an automatic referral.”*

Many of the critiques of family physicians’ capacity to work with addictions and mental health ultimately focused on their fee-for-service compensation model. Physician billing was discussed as a leading contributor to doctors not spending more time serving clients with mental health and addictions concerns in their family practice, thereby demotivating doctors from working with these clients. Other participants noted that family physicians may already feel like they are at capacity and do not have the ability to address their patients’ more time-intensive concerns. Participants also connected these factors with practices such as time-limited visits (e.g., 5 or 15 min) and no-show fees. Ultimately, participants described that fee-for-service acts as a barrier to addressing clients’ mental health and addictions concerns.

Psychiatrist: *“I think the management piece tends to be harder given the model that a lot of the family physicians are forced to practice under. I don’t think it’s a lack of capability, but I think it’s literally how what else they’re expected to do in their time that becomes the challenge and so this is the feedback I get from a lot of colleagues that might do family medicine.”*

AHS AMH Management: *“Physicians, I mean they’re paid fee-for-service so they need to, they want to see as many … they’re driven by the number of clients they see and the workload.”*

Despite barriers, participants, and especially physicians themselves, noted that family doctors already address mental health concerns on a regular basis in their practices. These individuals observed that many family physicians have strong relationships with their clients and are adept at addressing addictions and mental health concerns. Others noted that the family doctors they worked with were willing and eager to learn more about addiction and mental health.

PCN Management: *“The literature says 25–40% of all visits to family physicians involve a mental health complaint, so they must be handling that. I don’t think they can turn from it and run from it, but again, some find it a rewarding part of their practice and others prefer to have help. […] But I think the degree or the complexity of the mental health will decide whether it stays in the practice or it gets referred out, not different from cardiology or orthopedics or…”*

Psychiatrist: *“I think, in fairness to family docs, we see in the PCN that they come looking for help, but I think this should be a targeted area to deal with addictions. […] I see it reflected in what comes at the college level and how the college is trying to change attitudes, etc., but I also see it in my own experience in primary care.”*

#### 3.2.3. Lack of Addictions and Mental Health Capacity: “There Are Never Enough Psychiatrists”

While participants noted issues with family physician capacity, many participants noted that the limited capacity of psychiatrists (and less so, addiction and mental health therapists) also acts as a barrier to shared care.

Psychiatrist: *“Well if they want that, then they’ll have to wait more, though. If you want that, then instead of seeing me within two weeks, or within a month, it’ll be six months to a year. You know, that’s private practice. That’s then a six month or a year wait list.”*

Family Physician: *“In fact when people in other places have tried to remove that role from family physicians and put it all onto psychiatrists, the system’s just fallen completely flat because there are never enough psychiatrists, they can never spend enough time with all the patients, we cannot have that, you know, this isn’t something, ‘Oh, you’ve got an addiction, oh my god, go away and see a psychiatrist.’ It cannot be like that.”*

#### 3.2.4. Concerns with Information-Sharing: “Confidentiality Is a Problem”

Participants noted their concerns with confidentiality and liability within a shared care model. Many participants were apprehensive about what information would be shared and preferred limited information sharing, especially when discussing communication through electronic medical records. Participants cited concerns with confidentiality and varying interpretations of Alberta’s Health Information Act and Freedom of Information and Protection of Privacy Act as major barriers. Others indicated that sharing clients’ personal disclosures could negatively impact their relationships with their clients, given the stigma around addiction and mental health; they indicated that this informed their lack of information-sharing and their belief in the importance of confidentiality. Conversely, some participants advocated for less fear around confidentiality because of safety and having access to information in emergency situations.

Client: *“One thing I’d adjust on what I’ve said is just that maybe if it’s something that’s really confidential, really personal, maybe something like that you wouldn’t want shared between all three [therapist, psychiatrist, and family doctor]”.*

AHS AMH Management: *“I think that’s a real fundamental issue about confidentiality and how this information is going to be available and how is it recorded […] you know, because stigma is real. There’s a reason why people are worried about others knowing about their issues and that kind of thing, so how do we circumvent that so that there could be collaboration around the care without exposing the client?”*

Related legal barriers and benefits to sharing information were also noted. Many of these concerns centered on liability for medication management between the psychiatrist and family doctor when sharing responsibility for a client’s medications. On one hand, participants mentioned the benefits of communicating with each other about clients’ medications. Others, however, discussed the risks of care providers not having clear roles and responsibilities or noted that liability concerns may lead some practitioners to make unilateral decisions.

Client: *“Does shared care mean lack of individual responsibility?”*

AHS AMH Clinician: *“If you have so many different doctors trying to manage the same thing in different ways all at once, that’s risky.”*

PCN Management: *“I think the other key piece is you often hear, “Well at the end of the day I’m responsible so I’ll make the decision,” and even though it’s like, my license is on the line, or it’s—so I think there’s got to be, shared care is who’s the shared decision maker? Right? And that also has to be enabled through a whole bunch of other things. But you can only share care if you’re sharing decisions.”*

#### 3.2.5. Practitioner Buy-In: “This Is How Things Are Done, and We’ve Always Done It This Way”

Participants expressed that one of the major barriers to instituting a shared care model is a lack of healthcare provider buy-in. Participants attributed this to providers’ unwillingness to devote the time to shared care and their reluctance to ask for help and collaborate. While some participants noted that the family physicians, they worked with were eager to collaborate and practice shared care, others noted that some physicians were not comfortable working with patients’ addiction and mental health concerns and were disinclined to learn. Participants from AHS and PCNs noted barriers including territorialism, egos, skepticism about shared care as a model, differences in work structures and approaches, and an unwillingness to change.

AHS AMH Clinician: *“If you believe in shared care as a practice […] so physician buy-in, like your primary care provider buy-in, like really enables that.”*

AHS AMH Management: *“I think culture as well prevents it. There’s a whole lot of “this is how things are done, and we’ve always done it this way.” […] Absolutely culture for sure. […] There’d be some big change management.”*

### 3.3. Suggestions to Enable Shared Care

Many of the suggestions that participants supplied were related to improving current nonexistent or fragmented communication pathways between primary care and AHS addictions and mental health. This includes suggestions about Electronic Medical Record (EMR) communication, referrals, personal relationship building, and physical co-location.

#### 3.3.1. Electronic Medical Record (EMR) Communication: “That Online, Fluid Conversation”

Many participants suggested technology and EMR solutions to communication barriers, including having everyone, including primary care, connected to the same EMR. Participants noted that even having limited access to electronic information about clients’ access to services, care plan, and/or list of care providers could aid in shared care. The idea that communication pathways need to be standardized and efficient with consistent charting requirements and infrastructure was common across responses. Participants noted that standardized language and approaches need to accompany any technological solution. Participants also noted drawbacks to a shared EMR approach, including confidentiality issues, concerns with the feasibility of a shared EMR, and worries that passive solutions are not sufficient to establish truly collaborative shared care.

AHS AMH Clinicians: *“It would be nice if the primary care and all GPs were also on the same EMR as us, because they are so disconnected. They need us to be like reaching out constantly which takes effort and time, then they aren’t available, they aren’t there, they’re on vacation, the secretary doesn’t know, they don’t know anything about this client, there’s nothing in the file.”*

AHS AMH Management: *“And to me the biggest thing that prevents or disenables is getting rid of those silos, getting rid of the differences, like if you’re going to have primary care networks, then only bring them in, we should be on the same EMRs, we should be in the same locations, we should have hopefully letters of understanding that we can communicate freely or whatever’s required. All those barriers should be gone.”*

PCN Management: *“I’m looking at a variety of ways of doing that, of, right, not just old school like consultation letter faxed somewhere, into the oblivion, right, and then there’s an expectation that ginormous pile of other consultation letters that arrive at a physician’s desk every day, there’s a way to have that online, fluid conversation.”*

#### 3.3.2. Relationship Building and Collaboration: “Picking Up the Phone”

Many participants focused on how actively building relationships can enable shared care. The majority of participants who expressed positive views about current communication practices spoke about their informal, personal relationships with other clinics and programs. Participants noted that reaching out with phone calls, giving out contact information, and being available to educate and have conversations about their clients’ care were strategies that enabled shared care. Participants also described personal communication and regular conversations as key enablers of collaborative, team-based client care. However, they also noted that maintaining these relationships requires active effort. Some participants criticized relying solely on individual relationships, saying that developing personal relationships is not a standardized or long-term approach to shared care across large organizations and taking factors such as staff turnover into account.

AHS AMH Clinicians: *“I don’t think our clinic has an overall approach, but it’s all relationship based, based on who the therapist is, so I know from my own practice, I just have certain GPs and PCNs that I’ve built a relationship with over time, that we work beautifully together, we consult, we meet, if we’re trying to coordinate treatment or some kind of care, they’ll facilitate the medical component, and managing the withdrawal in the community without having to go to detox and all of those pieces, and so we do work in partnership, but it’s not an AHS partnership, it’s just an individual relationship based on […] my clients and the community”.*

PCN Therapist: *“But we have that relationship where we can phone up and say hey what’s going on like I need a consult or I need what’s happened last time with the psychiatrist and they’ll send it immediately.”*

Family doctor: *“What’s missing right now, so defining who your team is and then having the frequent ability to communicate with the team and chat with your mental health worker, your social worker, and your psychiatrist […] but that communication is for me what’s really lacking in all of this is that ability to keep going back and redefining the issue and then determining what the plan is going to be depending on what we’ve seen going on.”*

#### 3.3.3. Physical Co-Location: “You’re in the Same Space”

Many participants argued that co-location is a solution to the current disconnect between primary care and AHS addiction and mental health services. Participants described co-location as enabling the development of personal relationships and team-based cross-specialty learning and collaboration.

Some participants discussed specific visions for interdisciplinary clinics or medical hubs, while others described the success of the PCN model with embedded teams and built-in mental health support. Participants from AHS also suggested onsite general practitioners at mental health clinics, especially for clients who do not have a family doctor.

AHS AMH Management: *“[I]f you co-locate resources and include the patient in those conversations, most of these shared care principles would be achieved. It sounds easy, I know it’s not.”*

Family Doctor: *“I get more of that through my primary care medical home with my embedded social workers or my embedded therapist because we will talk back and forth and say, ‘okay you guys are working on this, this is the therapy piece you’re doing, this is what we’re going with medications, I’m going to reinforce your therapy strategies every time the patient comes in.’ And as long as I know what they are then I can help reinforce them and redirect them to your strategies and okay carry on. And I feel like that’s a lot more cohesive model for managing those patients in a collaborative way when you can actually have the discourse about it.”*

PCN Management: *“But I think the comfort level has certainly changed with providers, clinicians that are right in the family office, there’s lots of discussions or hallway conversations around specific patient issues so now their confidence level or perhaps their knowledge or resources has now increased because there’s that constant communication happening on a daily basis for them to be aware of what to do in the moment with those patients.”*

#### 3.3.4. Referrals and Service Information: “More Bridging […] so They’re Aware of the Services”

There were suggestions that may make referrals easier and reduce inappropriate referrals. These included having better information about AHS services available and not requiring formal referrals or paperwork. Certain participants stressed that anyone who can identify a mental health or addiction issue should be able to make referrals to psychiatrists, with some also arguing for more self-referrals. Strategies for increasing the quality of service information included a continually updated online directory of AHS programs and eligibility criteria.

Family Doctor: *“For me too another aspect is kind of a central access searchable form of what programs are available. So, I mean in my perfect world, I’d be able to go on [to a] central system, enter the words like depression, addictions, past trauma history and have some list of possibly appropriate programs show up where it’s like this might be a good match for your patient.”*

AHS AMH Clinician: *“And I think that that’s the other thing that’s really important is to not burden GPs with referrals and all sorts of paperwork that they need to do.”*

AHS AMH Management: *“But then we get a lot of referrals which are inappropriate because they’re not complex, and then it’s so our relationship is a lot of educating, so when we do get access to a GP or a PCN then we spend a lot of time trying to educate them in terms of appropriateness”.*

AHS AMH Management: *“But any mental health therapist that can identify a mental health or addiction needs to be able to refer to a psychiatrist.”*

Throughout the discussions about referrals, follow-ups, and re-referrals, most participants advocated for fluid transitions and openness between AHS and primary care and “a mutual understanding of the scope of services.” Others suggested a centralized place to get information about AHS addiction and mental health services would be beneficial.

AHS AMH Management: *“Yeah I think a reciprocal relationship where you can refer to us and we can refer back, or we can take on the care for a bit and then you know perhaps once they’re stabilized we can return them back the physician care with of course any support as needed”.*

## 4. Discussion

The literature is inconsistent with defining shared care [29]. Consequently, understanding and approaches to shared care may be contextual. Participants in this study associated an ideal model of shared care with patient-centered care, holistic approaches, and multidisciplinary teams. “Patient-centered care” is health care in which all health care decisions and quality measurements are centered around the individual’s specific health needs and desired health outcomes. This model sees patients as partners with their health care providers. Providers, on the other hand, manage patients’ healthcare not beyond a clinical perspective, but also from an emotional, mental, spiritual, social, and financial lens [39]. This perspective resonates with the literature and our conceptual model; however, the emphasis from our study participant was on patient centeredness. This point of emphasis may contrast with many other studies. For example, a literature review by Kelly and colleagues posit that shared care must be systematic, have a common goal, targeted at a condition or population, have an agreed clinical pathway, among others [29]. Most of our study participants agreed that patient-centered service is an important pillar in the shared care service. They emphasized that “sharing” is an important concept in healthcare as patients can actively participate in the decision-making process. Patient-centered care requires that patients and clinicians work together to select tests, treatments, management or support packages, and requires the healthcare provider to help explain the health care problem, present options, discuss the benefits and risks, clarify patient values and preferences, and discuss patient abilities and self-efficacy [40,41,42,43]. Whereas information asymmetry is a common feature in the healthcare, it is increasingly recognized that the patient’s voice is critical to optimum recovery, particularly in mental health care. Several healthcare reform commissions have recommended patient-centered service models, especially for patients with multiple comorbidities, in which the primary care team interact consistently with patients [44,45]. 

Perceived barriers to the implementation of an ideal shared care model included fragmented communication between primary and secondary healthcare providers, patient and family physician discomfort with discussing addiction and mental health, a lack of staff capacity, confidentiality issues, and practitioner buy-in. Participants also identified four main enablers to successful shared care, including implementing shared electronic medical record systems, improving communication and collaboration, physical co-location, and increasing practitioner awareness of appropriate referrals and services. While these barriers and enablers were identified in the context of Edmonton Zone, many of the factors identified align with previous research and our conceptual model. The literature also demonstrates and describes current examples of shared care models that incorporate some of the key principles that participants identified.

Previous research indicates that patients will preferentially seek the help of their family doctor for mental health concerns [2]. The established relationship between patients and their family practitioners is likely to aid in facilitation of shared care implementation, and was identified as a key positive influence on access to shared care in previous research [40]. 

Shared or collaborative care has been shown to significantly improve depression management and result in cost savings to the health system when introduced in the care of patients with diabetes and comorbid depression. However, shared care has not been shown to improve medical control of diabetes. This suggests that while collaborative care could improve depression care, medical practitioners may need to do more to support patients to achieve diabetes control by improving adherence and promoting skill development [4,46]. A number of clinical trials have highlighted the effectiveness of collaborative services [4]. These include:

- TEAMcare, where a nurse care manager and primary healthcare physician introduced a close supervised service to patients with depression and another medical health condition(s) over 12 months, with weekly supervision from a psychiatrist [45,47,48,49].
- Integrated illness management and recovery (I-IMR), where an I-IMR specialist provided an eight-week curriculum, containing two modules for medical and psychiatric health, which were tailored according to the individual needs [50].
- The Primary Care Access Referral and Evaluation (PCARE), which was introduced to people with comorbid severe mental illness, where nursing care managers played the pivotal role in patient management care, through advocacy, information providing and motivational interviewing [51].
- Life goal collaborative care (LGCC), where enhanced self-management control of risky behaviors in bipolar patients resulted in less manic symptoms and lower blood pressure among the intervention group who were offered four weeks of mental health specialist support followed by twelve months of care manager support [52].

Many study participants defined an ideal shared care model as holistic, involving a multi-disciplinary team. A few studies of holistic approaches support this argument. A study involving a holistic health intervention for people with cognitive impairment resulted in a significant improvement in memory and cognition [53]. For the geriatric community, where the “polypharmacy” concept exists and guidelines around the managing comorbidities collaboratively with patients and or their caregivers have been developed, holistic approaches have also been successful [54]. Organizational campaigns, such as the Mind/Body Health Campaign in the United States, have be used to raise awareness to mental health as an integral part of overall health [55]. Effective and understanding leadership is perceived as essential in providing and maintaining a successful holistic care in the health system [49].

Consistent with findings from previous studies [4,26], participants in our study expressed the benefits of co-locating general practitioners and mental healthcare providers to enhance shared care. Co-location has been shown to improve attendance rates and the reception of diverse preventative services, including colorectal and metabolic screening and better blood pressure control [4,26]. Face-to-face meetings have been described as necessary for implementation of the collaborative service with a better patient centered care [4,26]. 

When considering the relationship between family physicians and mental health care providers, it is important to reduce fragmentation and integrate mental health services [20]. Our study’s participants described fragmented communication between healthcare providers as an obstacle to collaborative care. This view is consistent with the literature, which notes multilayered communication barriers: between family doctors and hospitals, physical and mental health care, and across health and social care [56].

A successful shared care model involving co-location, conceptualized as a “Reversed Shared Care in Mental Health,” was studied in North York General Hospital in Toronto, Ontario. This model consisted of a primary care clinic embedded in the mental health department, providing care for patients who did not have a family physician [5]. In a different shared care model for Clozapine management, consumers reported a high rate of satisfaction with the service received from family physicians [40]. These shared care models are similar to the metabolic clinic embedded within a mental health community clinic in Edmonton, and which is run by family physicians who provide physical healthcare for patients on antipsychotic medications.

Our participants identified collaborative communication channels with other health care providers, including phone calls, e-mails, and face-to-face encounters, as a means to improve patient care and to supplement or facilitate formal mental health training programs for family physicians. Improved communication has been shown to lead to more confident physicians who have better knowledge and a lower referral rate [57]. 

One of the enablers for collaborative care that participants mentioned was shared electronic medical records (EMR). Shared EMR facilitates the sharing of information and has been described in the literature as “key” to the successful integration of services. Even so, multiple considerations, including the confidentiality of patient records and technical support issues, need to be taken into account when implementing EMR systems for addiction and mental health issues [56]. Consistent with the literature, our study participants identified that confidentiality could become an issue with shared EMR, especially when the scope of services are not clearly outlined and when health providers need to obtain consent from their patients in order to share health care information with other providers [9].

Financial considerations have been cited as a barrier to the implementation of shared mental health care in prior research; specifically, concerns were identified around primary care physicians’ inadequate reimbursement for mental health services [7]. Higher “no show” and cancellation rates for patients with mental illness also negatively impacts physicians’ income who are paid through a fee-for-service compensation model [9]. The removal of this significant barrier may help with physician buy-in for shared care. The concern with a fee-for-service model was echoed by our participants, who noted that it may de-motivate family physicians from participating in a shared care model.

Stigma and concerns around disclosing remain barriers to patients accessing mental health care from family physicians [34], particularly among marginalized populations such as sexual and gender minorities [36]. According to one study, family physicians’ negative stereotypes around mental illness negatively impact patient satisfaction [30]. In addition, our participants argued that patients may be concerned with family physicians’ skills, training level, or ability to provide appropriate addiction and mental health treatments. Given that therapeutic alliance and trust are key features in the success of mental health treatment modalities, patients’ negative perceptions are an important obstacle to shared care. In the same context, mental health care providers can lack confidence in their ability to manage common physical health problems in addiction and mental health patients [56]. Both mental health and primary care providers would benefit from training in managing patients with both comorbid mental health and physical comorbidities. This may address the scope and the limits of the service introduced by each healthcare provider [9,56]. 

Collaboration between family physicians and psychiatrists could be facilitated through a common protocol and accessible recording system [6]. For example, Edmonton Zone addiction and mental health services have recently introduced the Level of Care Utilization System (LOCUS), which determines level of care using six main parameters: Risk of Harm; Functional Status; Medical, Addictive and Psychiatric Co-morbidity; Recovery Environment; Treatment and Recovery History; and Engagement and Recovery Status [58]. Each parameter has a number of criteria to help determine the level of care. Usually, six levels are identified: Recovery Maintenance and Health Management, Low Intensity Community Based Services, High Intensity Community-Based Services, Medically Monitored Non-Residential Services, Medically Monitored Residential Services, and Medically Managed Residential Services. Accordingly, the required services and resources are determined either by the number of the needed personnel (nurses, physicians, etc.), or the type of personnel. It is expected that the LOCUS tool will be employed across all primary and secondary care services to enable the implementation of shared addiction and mental health care across the zone. 

The shared care model emphasizes the importance of integration, collaboration and the co-location of mental healthcare providers and family physicians. Models can range from minimal integration between psychiatrists and family physicians, with intermittent communication for referrals, to a fully collaborative, integrated system, in which psychiatrists, family physicians, and other practitioners form one team and provide an integrated “interdependent” service [59]. It would be a significant help for policymakers and stakeholders to use such an objective and consistent tool for planning and quality improvement for the healthcare system [58,60]. 

While our study can be a useful tool to inform the implementation of a shared care approach, we would like to highlight some limitations. First, being a qualitative focus group study involving limited number of participants, it is possible that different stakeholders within the Edmonton Zone could share different perspectives about shared care based on their own experiences. Second, given the workplace context of many of the focus groups and presence of other participants, some participants may have felt less comfortable sharing negative views. While our study results help illustrate a clear picture of some barriers and enablers of shared care, as well as suggestions for implementation, our results reflect the Edmonton context, and should be interpreted with that in mind. Further, patients were not as vocal as health care providers during the FGDs, despite facilitation in this direction. This may be because the topic was more related to service delivery than service utilization. Patients are not as likely to feel informed or feel empowered to discuss service delivery. These are the paradigms and perceived power dynamics that shared-care models seek to correct. We are hopeful that as this model gets increasingly adopted, a paradigm shift that empowers the patient as a collaborator in health care will become mainstay. Notwithstanding these limitations, our study provides valuable insights into stakeholders’ views of the ideal addiction and mental health shared care model, and identifies enablers and barriers to implementation in the Edmonton Zone. 

## 5. Conclusions

Participants in our study identified the holistic, team-based approach and patient-centered care as two important features of an ideal shared care model. Whereas the literature is inconsistent with what makes up shared care, our study contributes to filling this gap by providing empirical contextual knowledge from the perspective of stakeholders involved with service delivery. Given the knowledge that frontline health workers often exercise discretionary powers in implementation which may differ from that of the policymaker or theorist [61], our research is a useful piece of the puzzle to the effective implementation of shared care, particularly in Edmonton and similar contexts. Specifically, the findings from our study will be useful in bridging the gap between how shared care is perceived by researchers and policymakers, versus how the model may be perceived and implemented by frontline stakeholders. 

Fragmented communication between primary and secondary healthcare providers, lack of comfort on the part of both patients and family physicians with discussing addiction and mental health issues, human resources challenges, confidentiality issues, and practitioner buy-in were identified as barriers to the implementation of the shared care model. These barriers, however, do not invalidate the usefulness of shared care models. Contextual adaptations are critical to improving acceptability and effectiveness of shared care models.

Future evidence synthesis of stakeholders’ perspective on shared care models may provide broader inferences, which may have more generalizable knowledge beyond our study settings.

## Data Availability

All relevant data are available within the manuscript.
